# Therapy onset and characteristics associated with improvement following HABIT-ILE in infants with unilateral cerebral palsy: secondary analysis of a randomized controlled trial

**DOI:** 10.3389/fneur.2026.1882719

**Published:** 2026-10-27

**Authors:** Astrid Carton de Tournai, Enimie Herman, Daniela Ebner-Karestinos, Estelle Gathy, Rodrigo Araneda, Anne Renders, Célia De Clerck, Seyma Kilcioglu, Yves Vandermeeren, Yannick Bleyenheuft

**Affiliations:** 1 Institute of Neuroscience (IoNS), UCLouvain, Brussels, Belgium; 2 Exercise and Rehabilitation Science Institute, School of Physical Therapy, Faculty of Rehabilitation Science, Universidad Andres Bello, Santiago, Chile; 3 Neurology Department, Stroke Unit/Motor Learning Lab, CHU UCL Namur, Yvoir, Belgium; 4 Louvain Bionics, UCLouvain, Louvain-la-Neuve, Belgium; 5 Physical Medicine and Rehabilitation, Cliniques Universitaires Saint-Luc, Brussels, Belgium

**Keywords:** cerebral palsy, hemiplegia, rehabilitation, infant, treatment outcome, motor skills

## Abstract

**Aim:**

To explore the association between therapy onset and changes in motor and functional outcomes and identify characteristics associated with greater changes in infants with unilateral cerebral palsy (UCP) following Hand-Arm Bimanual Intensive Therapy-Including Lower Extremities (HABIT-ILE).

**Method:**

This study included 38 infants aged 6 to 20 months (mean = 14.4 ± 4.3 months) with UCP. All received 50 h of HABIT-ILE and were assessed pre-intervention and 3 months later. Outcomes included Mini-Assisting Hand Assessment (Mini-AHA; primary), Gross Motor Function Measure-66 (GMFM-66) and Canadian Occupational Performance Measure (COPM). Regression analyses explored changes in outcomes in relation to age at therapy onset, prematurity, type and side of lesion, and baseline motor abilities.

**Results:**

Age at therapy onset was not significantly associated with changes in Mini-AHA or COPM satisfaction scores. However, younger age was associated with larger changes in the GMFM-66 and the COPM performance scores. Regarding baseline characteristics associated with greater improvement, the baseline GMFM-66 score was associated with changes in GMFM-66.

**Interpretation:**

These exploratory analyses found that younger age at therapy initiation was associated with larger changes in GMFM-66 and COPM performance scores, suggesting that early therapy onset may be associated with greater improvements in gross motor and functional performance. Baseline gross motor abilities were also associated with changes following HABIT-ILE, suggesting that these characteristics may contribute to variability in observed changes and warrant further investigation in larger studies.

**Clinical trial registration:**

https://clinicaltrials.gov/, identifier NCT04698395.

## Highlights

- Earlier therapy onset was associated with greater GMFM-66 and COPM performance score changes.
- Baseline motor abilities appeared to be related to subsequent changes in gross motor function.
- These patterns warrant further investigation of therapy timing and individual clinical characteristics as potential factors influencing HABIT-ILE response.

## Introduction

Children with cerebral palsy (CP) present a heterogeneous pathophysiology depending on the type, localization, extent and onset of the brain lesion (1). This heterogeneity results in a large panel of motor impairments, which can differentially affect functional abilities across individuals, and may contribute to differences in response to rehabilitation. Identifying factors associated with greater observed changes may therefore help characterize which children show greater changes during early intensive intervention. Evidence suggests that some neurodevelopmental and clinical features may influence treatment responsiveness. For instance, Chamudot et al. (2) reported that infants with periventricular white matter (PWM) injuries exhibited greater motor improvements following intervention (3), while Sakzewski et al. (4) identified left-sided hemiplegia as a predictor of more favorable outcomes (4).

In recent years, early age at intervention has emerged as one of the most extensively studied predictors of treatment response (5). Experimental work suggests that the potential for adaptive reorganization following early brain injury may be greatest during the initial stages of development, when motor development is rapid and neural networks are undergoing substantial maturation, with this capacity becoming more constrained with age (6, 31, 32). Advances in the early detection of CP, combined with growing evidence of heightened neuroplasticity during the first 2 years of life, have supported the development of early, evidence-based interventions (7, 33). Among these interventions, Hand-Arm Bimanual Intensive Therapy—Including Lower Extremities [HABIT-ILE (8, 9)] has demonstrated positive outcomes in terms of motor abilities, notably by enhancing the use of the more affected hand in children from 6 to 59 months (10, 11). Our baby- and early HABIT-ILE randomized clinical trials (RCTs) suggested that the optimal window for maximizing the impact on hand function in children with unilateral CP (UCP) is between 6 and 36 months (10, 11). However, whether initiating therapy earlier within this period is associated with greater improvements remains unclear.

In addition to the timing of intervention, the substantial variability in individual response raises the question of whether specific baseline characteristics are associated with greater improvements. A growing body of evidence suggests that several baseline characteristics may influence treatment response in children with CP. These include: (i) the type of brain lesion, which is determined by the stage of neural development at the time of the insult, with various neurological structures being affected (12); (ii) prematurity, which is associated with specific patterns of brain development and neuropathology, particularly involving the developing white matter (13, 34); (iii) the lesion laterality, since hemispheric lateralization plays a role in motor control (14, 15); and (iv) the degree of motor impairment at baseline, which may constrain or facilitate the potential for functional gains. In addition to their potential influence on baseline motor development, these characteristics may reflect differences in neural organization and motor abilities that could influence the capacity to engage in and benefit from intensive motor learning. However, the existing literature identifying features associated with favorable outcomes after motor intervention in children with CP has primarily focused on school-aged children, with limited data on infants and toddlers. Furthermore, findings in (pre-) school-aged children are controversial. While some identify factors like brain lesion or baseline manual performance as predictors, others do not corroborate these findings (3, 4, 16, 17).

Therefore, using data from our baby-HABIT-ILE RCT, this study addressed two related questions:

1. Is younger age at therapy initiation associated with greater improvements in infants with UCP aged 6 to 20 months?
2. Which baseline characteristics are associated with greater improvements following HABIT-ILE?

We hypothesized that initiating therapy at an earlier age might result in larger changes and that there might be an association between the response to HABIT-ILE and the infants’ features.

## Materials and methods

### Participants

This study protocol was approved by the university’s ethical board (Comité d’éthique Hospitalo-Facultaire/UCLouvain, Brussels, Belgium; reference number: 2013/01MAR/069 B403201316810g) and the original RCT was prospectively registered in ClinicalTrials.gov (NCT04698395). All parents provided their written informed consent.

Forty-eight infants were recruited by A.C.d.T. and E.H. to participate in a baby HABIT-ILE protocol in 2021 and 2022 in Brussels, Belgium. Inclusion criteria were (1) infants aged 6 to 18 months (corrected age if preterm birth) at first assessment (T0), (2) at risk of or diagnosed with UCP, and (3) and able to comply with the needs of the testing. Exclusion criteria were (1) uncontrolled seizures, (2) botulinum toxin injections, orthopedic surgery, or specific intensive therapy within 6 months before and until the end of the study, (3) any Magnetic Resonance Imaging (MRI) contraindications and (4) severe visual or cognitive impairments preventing the child from being interested in simple games.

### Study design

The results in this study are derived from an RCT with a two-arm parallel-group design and 1:1 allocation. Infants were pair-matched randomized using a computer-generated randomization program by A.C.d.T., E.H., and D.E.-K. before the baseline assessment to either the immediate- (*n* = 24) or the delayed (*n* = 24) treatment group. For the present secondary analysis, data from all 48 infants were pooled, with assessments conducted immediately before and 3 months after HABIT-ILE used to examine changes during the intervention period. Because all infants received HABIT-ILE during this period, the pre- to post-intervention changes were analyzed across the entire sample. In addition, changes occurring during the 3-month period preceding HABIT-ILE in the delayed-treatment group were analyzed separately to characterize spontaneous evolution in the absence of the intervention (control period). Therapy sessions were performed between March 8, 2021, and June 17, 2022.

### Intervention

Both groups received the same intervention, baby HABIT-ILE, delivered in the same environment and by the same supervision team. As detailed in the study protocol, all infants participated in 50 h of intervention over a 2-week period (5 h per day excluding naps) (18). Baby HABIT-ILE, an adaptation of HABIT-ILE (8, 9), is an intensive intervention based on motor skill learning principles. It is delivered as day camps that provide constant stimulation of both upper and lower extremities, and posture.

### Outcome measures

The Mini-Assisting Hand Assessment (Mini-AHA), assessing the use of the more affected hand as an assisting hand in bimanual activities, was the primary outcome measure (19). It was videotaped and blindly scored by certified examiners masked to group allocation and timing of assessments.

The Gross Motor Function Measure-66 (GMFM-66), also videotaped and blindly scored, assesses gross motor function (20). The semi-structured interview of the Canadian Occupational Performance Measure (COPM) was used to define the functional goals with parents, who were asked to quantify the child’s performance and satisfaction in relation to the specified goals (21).

### Baseline characteristics associated with observed changes

Baseline characteristics were examined for their associations with changes observed during the intervention period. Prematurity (birth < 36 weeks of gestation), the brain lesion side (left/right), determined based on the clinically affected side and the lesion type (brain malformation (BM), PWML or grey matter lesion (GML) (12) were considered as fixed factors. Furthermore, pre-intervention Mini-AHA and GMFM-66 scores, reflecting baseline motor function, as well as the corrected age, were considered as covariates.

These variables were selected based on their established relevance in the neurodevelopmental literature and their reported associations with motor outcomes in children with CP (3, 4, 16, 22, 23). They are accessible early in life, clinically interpretable, and may reflect differences in neurological organization and motor function that are associated with subsequent changes during intensive motor intervention.

### Sample size

Our sample size calculation, based on a pilot study (24) and designed to detect a 7.2 AHA-logit difference between groups, indicated that 15 infants per group were needed (*α* = 0.05, 1 − *β* = 0.9). To account for potential dropouts and differences between AHA and Mini-AHA measures, we recruited 24 infants per group (18). This sample size was calculated for the primary outcomes of the RCT and was not specifically powered for the secondary analyses presented in the current study.

### Statistical analysis

Analyses were performed using IBM SPSS Statistics 29.0. To examine the association between age at therapy initiation and changes observed during the intervention period, a simple linear regression was employed, using the change scores observed in the entire sample of each dependent variable (Mini-AHA, GMFM-66, and COPM -performance and satisfaction-). The corrected age in months when receiving the intervention was considered as a covariable. Similar analyses were conducted using the delta of change observed during the control period in the delayed treatment group to document changes occurring under conditions of regular daily activities.

To examine associations between baseline characteristics and changes observed during the intervention period, a multiple linear regression was used with the delta of changes observed in the entire sample with assessment scores as the dependent variables. Prematurity, the lesion side and the lesion type were considered as fixed factors and the Mini-AHA units and GMFM-66 score at baseline as covariates. Given the small and uneven distribution of lesion types in the sample, findings related to lesion type were considered exploratory and interpreted with caution. Pairwise comparisons between lesion types were based on estimated marginal means, with Šidák adjustment applied to account for multiple comparisons within these pairwise analyses.

Potential ceiling effects were considered when interpreting changes in outcome scores. A 2-sided *p* < 0.05 was considered significant. The investigation of the influence of therapy onset was anticipated in the trial protocol (18); however, the statistical approach described above and the analyses of baseline characteristics were not prespecified and were defined after completion of the RCT. All analyses should therefore be considered exploratory (*post hoc*). Accordingly, no adjustment for multiple comparisons was applied across the overall set of exploratory analyses.

## Results

The results of the RCT involving the children included in this article have been published elsewhere (11). Importantly, the sample size of the present secondary analysis was not determined based on its specific objectives or outcomes, but rather on the sample size calculation for the primary outcomes of the original RCT. Therefore, the analyses presented here should be considered exploratory and descriptive. The observed associations and patterns should not be interpreted as definitive predictors of treatment response or as evidence of causal effects of the intervention.

### Characteristics of the study population

Of the 48 infants included in the primary analysis of the RCT (11, see Table 1), 38 infants were included in this secondary analysis, having successfully completed the two key assessments required (pre-intervention and 3-month post-intervention) (8 dropped out, and 2 were excluded from analysis because they did not participate sufficiently in the assessments, possibly due to limited engagement associated with cognitive impairments, rendering their data invalid according to the blinded examiners, Flow Diagram, Figure 1; Supplementary Table S1). Finally, one infant from the delayed treatment group did not complete the Mini-AHA assessment, as he refused to participate in the test during all three assessment periods, despite having the motor abilities required to perform the tasks. As a result, his data were excluded from the analysis of characteristics associated with changes during HABIT-ILE. The pre-camp characteristics of the 38 infants included in the analyses reported in this paper are presented in Supplementary Table S1. Supplementary Table S2 provides descriptive statistics (mean and SD) for the outcome measures at pre-camp and post-camp for each treatment group and the total sample.

**Table 1:** Characteristics of the participants at baseline (T0), *n* = 48.

Characteristics	Categories	Immediate treatment group (*n* = 24)	Delayed treatment group (*n* = 24)	Total (*n* = 48)
Age, CA	Months (mean ± SD)	13.37 ± 4.32	13.00 ± 4.13	13.19 ± 4.19
	Range	6–19	7–19	6–19
Gender, No. (%)	Male/female	13 (54)/11 (46)	16 (67)/8 (33)	29 (60)/19 (40)
Lesion side, No. (%)	Right/left	11 (46)/13 (54)	11 (46)/13 (54)	22 (46)/26 (54)
Lesion type, No. (%)	Brain maldevelopments	2 (8)	3 (12.5)	5 (10)
	Periventricular lesions	5 (21)	3 (12.5)	8 (17)
	Grey matter lesions	17 (71)	18 (75)	35 (73)
Prematurity, No. (%)	<36 weeks/≥36 weeks	7 (29)/17 (71)	7 (29)/17 (71)	14 (29)/34 (71)
GMFCS-ER, No. (%)	I	5 (21)	4 (17)	9 (19)
	II	7 (29)	7 (29)	14 (29)
	III	6 (25)	6 (25)	12 (25)
	IV	5 (21)	4 (17)	9 (19)
	V	1 (4)	3 (12)	4 (8)
Mini-MACS, No. (%)	I	5 (21)	7 (29)	12 (25)
	II	9 (37)	4 (17)	13 (27)
	III	8 (33)	8 (33)	16 (33)
	IV	2 (8)	3 (12)	5 (10)
	V	0	2 (8)	2 (4)

CA, corrected age; GMFCS-ER, gross motor function classification system-expanded and revised (under 2 years old); Mini-MACS, manual ability classification system for children aged 1–4 years (scored by clinicians).

**Figure 1:**
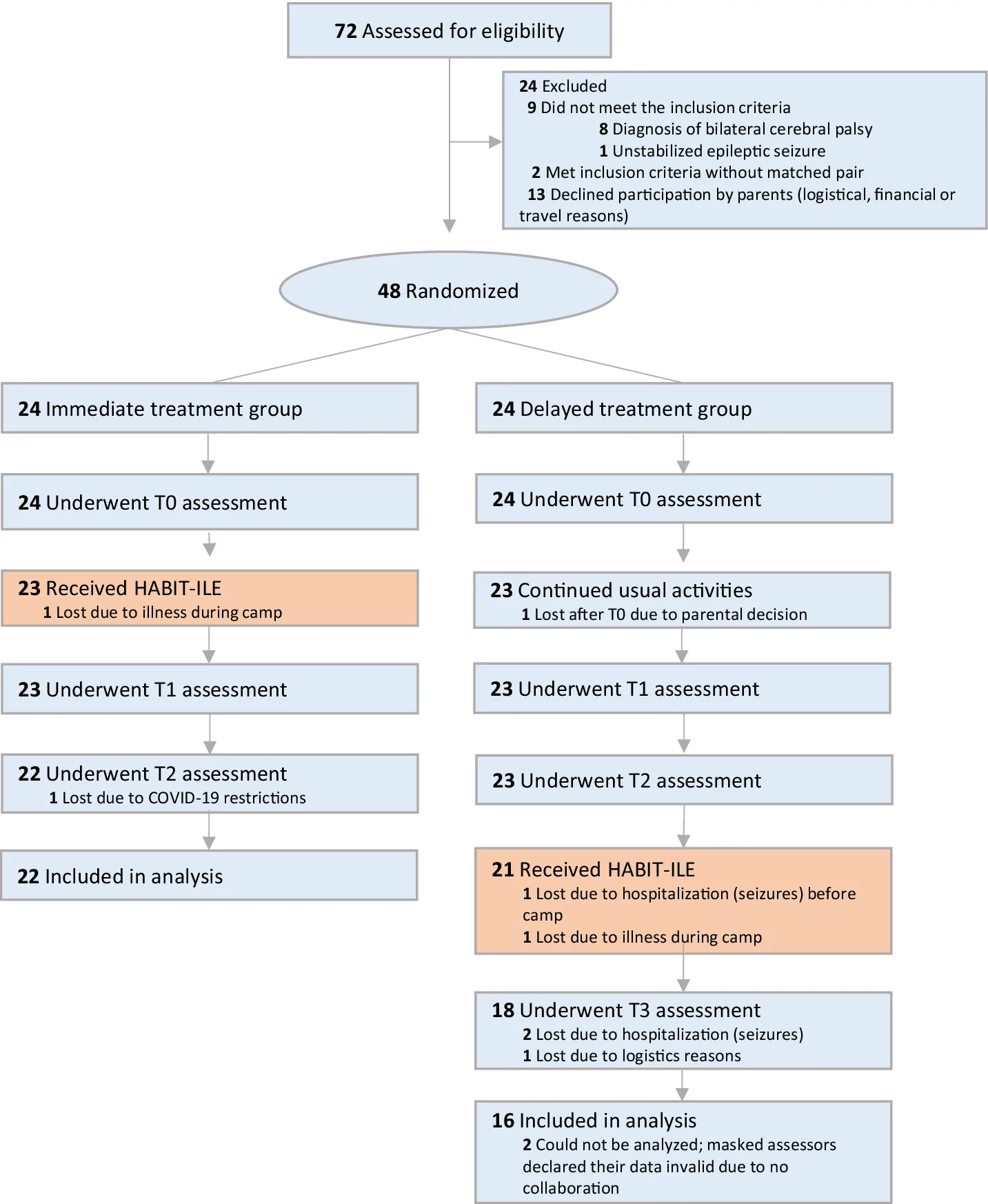
Flow diagram of participants. HABIT-ILE, hand-arm bimanual intensive therapy including lower extremities; T0, first assessment time (baseline); T1, second assessment time 1 month after first assessment; and T2, third assessment time 3 months after first assessment.

### Age at therapy onset and changes in outcome measures

For the Mini-AHA, no significant association between age at therapy onset and changes in scores was observed (*n* = 37, β regression coefficient = 0.21, 95% CI [−0.41, 0.83], *p* = 0.49; Figure 2a). To investigate whether the Mini-AHA score changes with age in the absence of specific therapeutic intervention, complementary analyses were conducted within the control period: no significant association between age and changes in Mini-AHA scores was observed during this control period (*n* = 16, *β* = −0.06, 95% CI [−1.04, 0.92], *p* = 0.89; Figure 2b).

**Figure 2:**
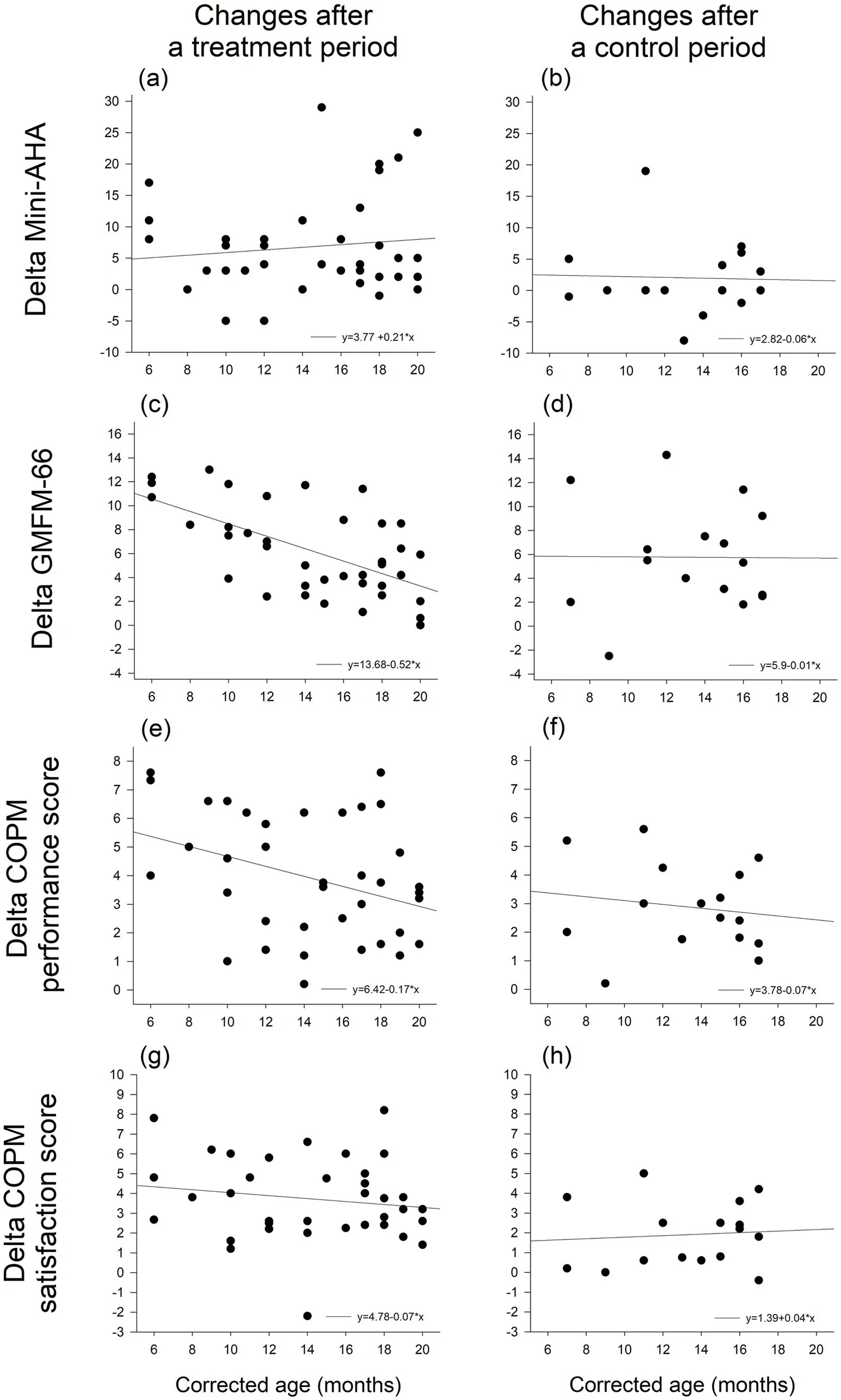
Simple linear regressions illustrating the relationship between age at therapy onset and clinical changes. The figure displays changes following a treatment period (baby HABIT-ILE) (left panels: a, c, e, g) compared to control period (right panels: b, d, f, h) for: **(a,b)** Mini-AHA, **(c,d)** GMFM-66, **(e,f)** COPM performance scores, and **(g,h)** COPM satisfaction scores. Each dot represents an individual participant. The solid lines represent the linear fitting lines.

Regarding gross motor function, age at therapy initiation was negatively associated with changes in the GMFM-66 after the intervention (*n* = 38, *β* = −0.52, 95% CI [−0.75, −0.29], *p* < 0.001; Figure 2c), with younger age at intervention initiation associated with greater observed changes. Complementary analyses within the control period showed no significant association between age and changes in the GMFM-66 (*n* = 16, *β* = −0.01, 95% CI [−0.74, 0.72], *p* = 0.97; Figure 2d).

For the COPM performance score, age at therapy initiation was negatively associated with changes in COPM performance scores during the intervention period (*n* = 38, *β* = −0.17, 95% CI [−0.33, −0.02], *p* = 0.03; Figure 2e), with younger age at intervention initiation associated with greater observed changes. Complementary analyses within the control period highlighted no significant association between age and changes in the COPM performance score (*n* = 16, *β* = −0.07, 95% CI [−0.32, 0.18], *p* = 0.57; Figure 2f). Regarding the changes in the satisfaction score, no significant association with age was observed after the HABIT-ILE intervention (*n* = 38, *β* = −0.07, 95% CI [−0.23, 0.08], *p* = 0.34; Figure 2g) or after the control period (*n* = 16, *β* = 0.04, 95% CI [−0.23, 0.31], *p* = 0.76; Figure 2h).

### Baseline characteristics associated with changes during HABIT-ILE

The multiple linear regression model used was based on the following variables: prematurity, lesion side, lesion type, baseline Mini-AHA units and baselineGMFM-66 score (*n* = 37) to examine whether those baseline characteristics were associated with changes in outcome measures. The regression coefficients reported below represent adjusted associations, with each predictor evaluated while controlling for all other predictors and covariates included in the respective model. The corresponding unadjusted associations are reported in Supplementary Table S3.

Regarding changes in the Mini-AHA scores, the model was significant (*F* = 2.66, *p* = 0.03, *R*^2^ = 0.35, adjusted *R*^2^ = 0.22). The exploratory analysis suggested an association between lesion type and change in Mini-AHA scores (*F* = 6.66, *p* = 0.004; Table 2), with larger estimated changes in infants with PWML (estimated marginal mean (EMM) = 16.25, SE = 2.92) in comparison with infants presenting with GML (EMM = 3.08, SE = 1.82, corresponding to an adjusted between-group difference of *β* = 13.18, 95% CI [3.5, 22.9], *p* = 0.005). However, given the small and uneven distribution of lesion types in the sample, these findings should be interpreted cautiously and considered exploratory.

**Table 2:** Multiple linear regression analysis with changes in Mini-AHA, GMFM-66 and COPM performance and satisfaction scores as dependent variables; prematurity, lesion type and lesion side as fixed factors; and the baseline Mini-AHA and GMFM-66 scores as covariates.

Model terms	Delta Mini-AHA				Delta GMFM-66				Delta COPM performance				Delta COPM satisfaction			
	*β*	SE	*p*-value	95% CI	*β*	SE	*p*-value	95% CI	*β*	SE	*p*-value	95% CI	*β*	SE	*p*-value	95% CI
Model *R*^2^/R^2^ adjusted			**0.034** 0.35/0.22				**0.003** 0.46/0.36				0.26 0.21/0.06				0.13 0.27/0.12	
Prematurity (non-prema/prema)	3.08	3.42	0.38	[−3.9; 10.1]	0.13	1.45	0.93	[−2.8; 3.1]	−0.63	1.01	0.54	[−2.7; 1.4]	0.30	0.94	0.75	[−1.6; 2.2]
Lesion type			**0.004**				0.36				0.63				0.11	
BM-PWML	−7.88	5.39	0.39	[−21.5; 5.8]	−1.61	2.29	0.86	[−7.4; 4.2]	−1.33	1.60	0.80	[−5.4; 2.7]	−3.15	1.47	0.12	[−6.9; 0.6]
PWML-GML	13.18	3.83	**0.005**	[3.5; 22.9]	−0.71	1.62	0.96	[−4.8; 3.4]	0.23	1.13	>0.99	[−2.6; 3.1]	1.77	1.05	0.27	[−0.9; 4.4]
BM-GML	5.29	3.94	0.47	[−4.7; 15.2]	−2.33	1.67	0.43	[−6.5; 1.9]	−1.1	1.17	0.73	[−4.0; 1.8]	−1.38	1.07	0.50	[−4.1; 1.3]
Lesion side (right/left)	0.99	2.36	0.68	[−3.8; 5.8]	2.03	1.00	0.051	[−0.01; 4.1]	−0.05	0.70	0.94	[−1.5; 1.4]	−0.27	0.64	0.68	[−1.6; 1.0]
Baseline Mini-AHA unit	−0.04	0.06	0.49	[−0.16; 0.08]	0.03	0.02	0.29	[−0.02; 0.08]	0.03	0.02	0.09	[−0.01; 0.06]	0.03	0.02	0.08	[−0.004; 0.06]
Baseline GMFM-66 score	0.12	0.13	0.34	[−0.1; 0.4]	−0.23	0.05	**<0.001**	[−0.34; −0.12]	−0.10	0.04	**0.018**	[−0.17; −0.02]	−0.08	0.04	**0.032**	[−0.15; −0.01]

BM, brain malformation; PWML, periventricular white matter lesion; GML, grey matter lesion; *β,* unstandardized regression coefficient; *SE*, standard error of the coefficient; *CI*, confidence interval; *R*^2^, coefficient of determination. For lesion type, values are pairwise differences between estimated marginal means; *p*-values and 95% CIs are Šidák-adjusted for multiple comparisons. Bold values indicate statistical significance (*p* < 0.05).

Prematurity, lesion side, baseline Mini-AHA units and baseline GMFM-66 score were not significantly associated with changes in Mini-AHA scores following the HABIT-ILE intervention (*p* = 0.38, *p* = 0.68, *p* = 0.49 and *p* = 0.34; Table 2).

Regarding GMFM-66, the model was found to be significant (*F* = 4.33, *p* = 0.003, *R*^2^ = 0.46, adjusted *R*^2^ = 0.36). Baseline GMFM-66 score was significantly associated with change in GMFM-66 (*F* = 17.45, *p* < 0.001, Table 2), with lower baseline GMFM-66 scores being associated with greater observed increases in GMFM-66 scores during the intervention period (*β* = −0.23, 95% CI [−0.34, −0.12]; Figure 3). A borderline significant association was observed between lesion side and GMFM-66 changes (*F* = 4.12, *p* = 0.051), with infants presenting a right brain lesion (EMM = 6.72, SE = 0.94) showing a tendency toward greater observed changes compared to infants with a left brain lesion (EMM = 4.69, SE = 0.79, *β* = 2.03, 95% CI [−0.01, 4.10]).

**Figure 3:**
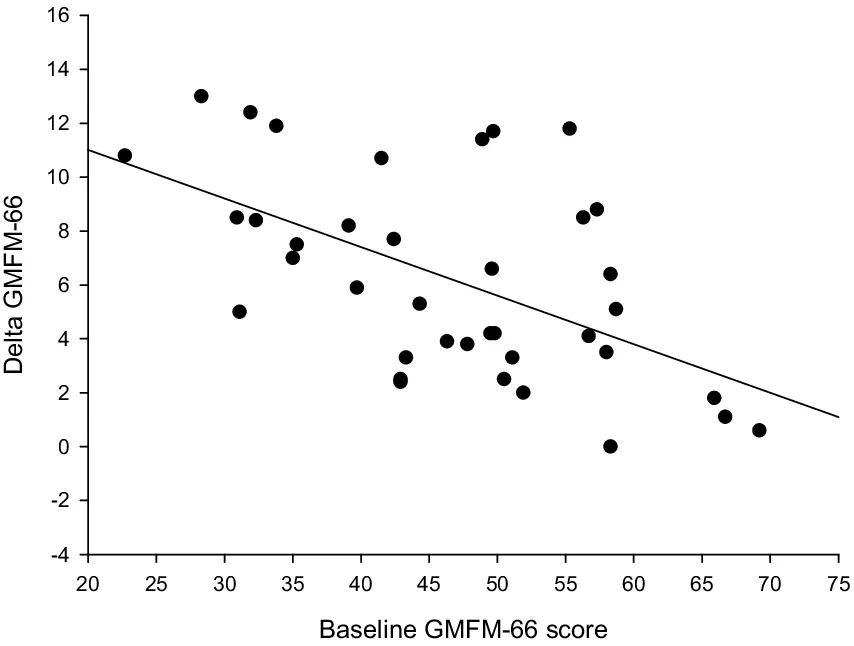
Linear regression between changes in GMFM-66 score after HABIT-ILE and the baseline GMFM-66 score.

For the COPM, the model was non-significant regarding the performance score (*F* = 1.36, *p* = 0.26, *R*^2^ = 0.21, adjusted *R*^2^ = 0.06). Nevertheless, within the model, baseline GMFM-66 score was significantly associated with changes in the COPM performance score (*F* = 6.30, *p* = 0.018; Table 2), suggesting that infants with lower baseline GMFM-66 scores exhibited larger changes in the COPM performance score than infants with higher baseline GMFM-66 scores (*β* = −0.10, 95% CI [−0.17, −0.02]). The same pattern was observed for the satisfaction score, with a non-significant model (*F* = 1.84, *p* = 0.13, *R*^2^ = 0.27, adjusted *R*^2^ = 0.12) and a significant association between COPM satisfaction score and baseline GMFM-66 (*F* = 5.08, *p* = 0.032, Table 2), favoring infants with poorer baseline GMFM-66 score (*β* = −0.08, 95% CI [−0.15, −0.01]).

## Discussion

The main observations from this exploratory analysis were that the age at therapy onset, within the study’s age range (6–20 months), showed no statistically significant association with observed changes in the Mini-AHA and the COPM satisfaction score in this sample. However, age at therapy onset was associated with changes in the GMFM-66 and the COPM performance scores: the infants who received therapy earlier in their development demonstrated larger changes compared to those who received the intervention later. Regarding baseline characteristics associated with changes during HABIT-ILE, infants with lower baseline gross motor function demonstrated larger changes in GMFM-66 and COPM (performance and satisfaction) scores after the intervention than those with a higher gross motor function at baseline.

### Age at therapy onset

For the Mini-AHA, no statistically significant association between age at therapy onset and observed changes in scores was detected in this sample. This observation is not in line with our initial assumption, which was based on neuroplasticity literature suggesting that earlier therapy onset might result in larger improvements in hand function. According to the neurodevelopmental plasticity literature, the window of opportunity to prevent the consequences of secondary neuroplastic damage in CP is suggested to fall within the first 12 to 24 months of life (6, 25). However, the upper limb findings from Araneda et al. (10), which demonstrated differential responses in children younger and older than 36 months, suggest that this window may extend beyond the initial 12 to 24 months, potentially reaching up to 36 months post-term (10). This discrepancy between our findings (no statistically significant association between the timing of therapy onset and changes in manual ability) and those of Araneda et al. (10) may, in part, be explained by the different assessments used: the Mini-AHA in our study versus the AHA in Araneda et al. (10). The Mini-AHA has shown no association between age and ability scores in infants aged 8 to 18 months, indicating that it captures performance rather than maturational change (19). In contrast, the AHA has documented developmental progress in the use of the affected hand from 18 months to 6 years (26).

The results related to the GMFM-66 indicate larger changes when the intervention was introduced earlier, potentially supporting the hypothesis that earlier intervention may be associated with larger changes in gross motor function. This suggests that the magnitude of observed changes in gross motor function might decrease with age, particularly between 6 and 20 months. Although this age-related pattern was not observed during the control period, considerable inter-individual variability in GMFM-66 changes was observed, particularly during the control period, where the sample size was limited. The larger changes observed in younger infants may partly reflect their developmental stage, as younger infants generally had fewer gross motor skills at baseline and therefore greater potential for the acquisition of new skills over time. Given the relatively small sample size, particularly during the control period, these analyses cannot determine whether the observed age-related pattern reflects differential changes associated with HABIT-ILE, natural developmental variability, or a combination of both. Two additional considerations are relevant when interpreting the age-related changes in GMFM-66. First, although the GMFM-66 is widely regarded as a valid measure, its use in this specific population (0–3 years old) has typically treated the age group as a homogeneous entity (27). No research has specifically examined whether the sensitivity or responsiveness of the GMFM-66 differs between young infants (e.g., 6–12 months) and toddlers (2–3 years), and the number of applicable test items likely differs between these age groups. Second, while substantial changes were observed, it is notable that most infants exceeded the minimal clinically important difference (MCID) for the GMFM-66, even among the older children. Similarly, Araneda et al. (10) reported large GMFM-66 gains in toddlers aged 1.5 to 5 years. This indicates that the impact of the intervention is not limited to the youngest infants, as substantial improvements continue to be observed beyond infancy.

Overall, these results suggest that within the time window from 6 to 20 months post-term, the therapy onset time may be associated with changes in gross and functional motor outcomes, whereas no statistically significant association was detected with changes in the use of the more affected hand in bimanual activities in this sample. This difference across outcomes might reflect the distinct maturation timelines of neural pathways governing gross motor and upper limb functions, with the former exhibiting heightened neuroplasticity during this developmental period (28, 29).

### Characteristics associated with changes during HABIT-ILE

Regarding characteristics associated with changes in the use of the more affected hand (quantified by the Mini-AHA), infants seem to improve their upper limb function, regardless of their baseline manual performance. This observation is in line with those reported by Chamudot et al. (2) in infants following modified constraint induced movement therapy (CIMT) or bimanual training (2), but does not support our hypothesis that poorer manual performance would be associated with larger changes during the intervention period. Our assumption was based on the findings in preschool and school aged children with UCP, showing that children with poorer hand function benefited the most from upper limb interventions (4, 16). The discrepancy in findings may be partly explained by age differences between the samples. Although no statistically significant association between age at therapy onset and observed changes in Mini-AHA scores was detected in this sample within the 6- to 20-month age range, this does not rule out a potential influence of age. Alternatively, the Mini-AHA and AHA may not follow the same developmental trajectory, or their sensitivity to change may differ. On the other hand, regarding lesion type, the exploratory analysis suggested a potential association between lesion type and changes in Mini-AHA scores. However, the highly uneven distribution of lesion types in our sample substantially limits the interpretation of this finding, which should be considered hypothesis-generating and requires confirmation in larger, more balanced samples. This is consistent with previous findings highlighting an association between lesion type and efficacy of upper limb treatment in infants (3). However, studies involving school-age children with UCP did not report a similar relationship between functional changes and brain lesion characteristics (17). These differing findings highlight the need for further investigation of lesion type as a potential factor contributing to variability in observed changes during early development.

A further descriptive observation was that changes in gross motor function were associated with initial severity of motor impairment. Based on the results of the GMFM-66 and the COPM performance scores, infants with the lowest baseline motor function seemed to demonstrate the largest changes in gross motor function. A possible explanation is the moderate positive correlation between the infants’ age and their baseline GMFM-66 score (*r* = 0.56, *p* < 0.001 for this study). Younger children, who presented with lower GMFM-66 scores, might have been more likely to exhibit larger changes, as discussed in the section on therapy onset. An alternative explanation might be that infants with higher gross motor function follow developmental curves closer to those of typically developing children, and their improvements might be constrained by a ceiling effect, driven by the natural age-related maturation of their neurological (30) and musculo-skeletal systems.

The potential for ceiling effects was also considered. No participant had a maximum GMFM-66 score at baseline, and the two participants with relatively high baseline Mini-AHA scores (>90 logit-based units) did not reach the maximum score at the 3-month follow-up. For the COPM, goals with a baseline score of 10/10 (i.e., no difficulty performing the activity or complete satisfaction) were not selected as treatment goals. Therefore, substantial ceiling effects were unlikely to have meaningfully influenced the observed changes in this sample.

### Limitations

Although this study has a relatively large sample size compared to similar previously published studies, the sample size was determined for the primary outcomes and was not powered for the present secondary analyses. Some subcategories were also underrepresented, limiting the precision and generalizability of the results. Therefore, these findings should be considered descriptive and hypothesis-generating rather than conclusive, and interpreted in the context of natural developmental variability.

## Conclusion

In this exploratory study, no statistically significant association was detected between age at therapy onset and observed changes in Mini-AHA scores within the 6- to 20-month age range in this sample. Furthermore, baseline gross motor function was associated with observed changes during the intervention period. Specifically, infants with lower baseline gross motor function showed larger changes in GMFM-66 and COPM scores. There is a need for studies that identify characteristics associated with greater observed changes following other (early) interventions to pool results in meta-analyses and achieve evidence from larger sample sizes. This approach would facilitate broader generalization of the findings and enable more tailored rehabilitation strategies for each child according to his/her own specificities.

## Data Availability

The raw data supporting the conclusions of this article will be made available by the authors, without undue reservation.
