# Supplementary material for: Therapy onset and characteristics associated with improvement following HABIT-ILE in infants with unilateral cerebral palsy: secondary analysis of a randomized controlled trial
Source: Front Neurol. 2026 Oct 27;17:1882719. doi: 10.3389/fneur.2026.1882719 (PMC13761358; doi:10.3389/fneur.2026.1882719)
Supplement: Supplementary file 1 [file Supplementary_file_1.docx]

**Supplementary material**

**Supplementary Table S1.** Pre-camp baseline characteristics of the 38 participants included in the secondary analysis, according to original treatment allocation

| Characteristics | Categories | Immediate treatment group (n=22) | Delayed treatment group (n=16) | Total  (n=38) |
| --- | --- | --- | --- | --- |
| Age, CA | Months (Mean ± SD) | 13.00 ± 4.34 | 16.31 ± 3.49 | 14.39 ± 4.29 |
|  | Range | 6-19 | 10-20 | 6-20 |
| Gender, No. (%) | Male / Female | 11 (50) /11 (50) | 10 (62) /6 (38) | 21 (55) /17 (45) |
| Lesion side, No. (%) | Right / Left | 9 (41)/13 (59) | 8 (50)/8 (50) | 17 (45)/21 (55) |
| Lesion type, No. (%) | Brain maldevelopments | 2 (9) | 2 (12,5) | 4 (11) |
|  | Periventricular lesions | 5 (23) | 2 (12,5) | 7 (18) |
|  | Grey matter lesions | 15 (68) | 12 (75) | 27 (71) |
| Prematurity, No. (%) | <36 weeks/ ⩾36 weeks | 6 (27)/16 (73) | 5 (31)/11 (69) | 11 (29)/27 (71) |
| GMFCS-ER, No. (%) | I | 4 (18) | 4 (25) | 8 (21) |
|  | II | 6 (27) | 7 (44) | 13 (34) |
|  | III | 6 (27) | 3 (19) | 9 (24) |
|  | IV | 5 (23) | 2 (12) | 7 (18) |
|  | V | 1 (5) | 0 | 1 (3) |
| Mini- MACS, No. (%) | I | 4 (18) | 3 (19) | 5 (13) |
|  | II | 8 (36) | 5 (31) | 13 (34) |
|  | III | 8 (36) | 5 (31) | 13 (34) |
|  | IV | 2 (9) | 3 (19) | 5 (13) |
|  | V | 0 | 0 | 0 |

Abbreviations: CA, corrected age; GMFCS-ER, Gross Motor Function Classification System-Expanded and Revised (under 2 years old); Mini-MACS, Manual Ability Classification System for children aged 1-4 years (scored by clinicians).

**Supplementary Table S2.** Descriptive statistics of outcome measures before and after HABIT-ILE according to treatment group

|  | Pre-camp | Post-camp |
| --- | --- | --- |
| Mini-AHA, Mini-AHA-units |  |  |
| Immediate treatment group (n=22) | 42.09 (24.5) | 49.27 (26.6) |
| Delayed treatment group (n=15) | 51.60 (27.5) | 57.56 (26.3) |
| Total (n=37) | 45.95 (25.8) | 52.76 (26.4) |
| GMFM-66, logits % |  |  |
| Immediate treatment group (n=22) | 43.9 (9.4) | 50.47 (8.3) |
| Delayed treatment group (n=16) | 50.5 (12.9) | 56.16 (11.0) |
| Total (n=38) | 46.7 (11.3) | 52.87 (9.8) |
| COPM Children’s Performance, Mean/10 |  |  |
| Immediate treatment group (n=22) | 2.35 (0.9) | 7.34 (1.7) |
| Delayed treatment group (n=16) | 5.18 (1.7) | 7.59 (1.5) |
| Total (n=38) | 3.54 (1.9) | 7.45 (1.6) |
| COPM Parent’s Satisfaction, Mean/10 |  |  |
| Immediate treatment group (n=22) | 2.95 (1.3) | 7.37 (1.6) |
| Delayed treatment group (n=16) | 5.08 (1.6) | 7.81 (1.5) |
| Total (n=38) | 3.85 (2.0) | 7.56 (1.55) |

Values are presented as mean (SD). Pre-camp and post-camp values are reported for the immediate and delayed treatment groups and for the total sample. The sample size for Mini-AHA differs because one participant did not have complete Mini-AHA data at both assessment time points. Abbreviations: AHA, Assisting Hand Assessment; GMFM-66, Gross Motor Function Measure; COPM, Canadian Occupational Performance Measure

**Supplementary Table S3.** Unadjusted associations between baseline characteristics and changes in outcome measures

| **Predictor** | **Δ Mini-AHA** | **Δ GMFM-66** | **Δ COPM performance** | **Δ COPM satisfaction** |
| --- | --- | --- | --- | --- |
| Baseline Mini-AHA | β = -0.01 \| SE = 0.05 \| p = .85 \| 95% CI [-0,12 , 0.10] | β = -0.03 \| SE = 0.02 \| p = .24 \| 95% CI [-0,08 , 0.02] | β = 0.005 \| SE = 0.014 \| p = .70 \| 95% CI [-0,02 , 0.03] | β = 0.009 \| SE = 0.013 \| p = .50 \| 95% CI [-0,02 , 0.04] |
| Baseline GMFM-66 | β = 0.06 \| SE = 0.12 \| p = .63 \| 95% CI [-0,18 , 0.29] | β = -0.18 \| SE = 0.05 \| **p < .001** \| 95% CI [-0,27 , -0.09] | β = -0.05 \| SE = 0.03 \| p = .10 \| 95% CI [-0,11 , 0.01] | β = -0.034 \| SE = 0.03 \| p = .26 \| 95% CI [-0,09 , 0.03] |
| Prematurity | β = 3.55 \| SE = 2.92 \| p = .23 \| 95% CI [-2.37 , 9.47] | β = -0.72 \| SE = 1.33 \| p = .59 \| 95% CI [-3.43 , 1.98] | β = 0.66 \| SE = 0.76 \| p = .40 \| 95% CI [-0,89 , 2.21] | β = 0.72 \| SE = 0.73 \| p = .33 \| 95% CI [-0,75 , 2.19] |
| Lesion type | **p = .015** | p = .44 | p = .85 | p = .94 |
| Lesion side | β = -0.35 \| SE = 2.65 \| p = .90 \| 95% CI [-5.73 , 5.03] | β = -2.26 \| SE = 1.16 \| p = .06 \| 95% CI [-4.61, 0.10] | β = -0.12 \| SE = 0.70 \| p = .87 \| 95% CI [-1,54 , 1.31] | β = 0.07 \| SE = 0.67 \| p = .91 \| 95% CI [-1,29 , 1.43] |

β, unstandardized regression coefficient, SE, standard error, CI, confidence interval. For lesion type, p values represent the overall unadjusted association across the three lesion types.
